# Influence of Core and Membrane Composition on Drug Release from MCC/Isomalt-Based Matrix Pellets in Biorelevant Osmolarity Media

**DOI:** 10.3390/pharmaceutics18091046

**Published:** 2026-08-23

**Authors:** Christian Fleck, Isameddin Aghrbi, Kristina Vlahovic, Franciska Erdő, András József Laki, Nikolett Kállai-Szabó, István Antal, Miléna Lengyel

**Affiliations:** 1 Department of Pharmaceutics, Semmelweis University, Hőgyes Endre Street 7, 1092 Budapest, Hungary; christian.petszulat@stud.semmelweis.hu (C.F.); aessameddin@yahoo.com (I.A.); vlahovic.kristina@phd.semmelweis.hu (K.V.); kallai.nikolett@semmelweis.hu (N.K.-S.); 2 Faculty of Information Technology and Bionics, Pázmány Péter Catholic University, Práter Street 50/A, 1083 Budapest, Hungary; erdo.franciska@itk.ppke.hu (F.E.); laki.andras1@semmelweis.hu (A.J.L.); 3 Department of Biophysics and Radiation Biology, Semmelweis University, Tűzoltó Street 37-47., 1094 Budapest, Hungary; 4 Center for Pharmacology and Drug Research & Development, Semmelweis University, Üllői Street 26., 1085 Budapest, Hungary

**Keywords:** matrix pellet, functional core, MCC, isomalt, ibuprofen sodium, osmolarity-independent release, polymethacrylate copolymer, controlled-release, experimental design, formulation study

## Abstract

**Background/Objectives**: Matrix pellet formulations enable homogeneous incorporation of the active ingredient and advanced control of release depending on the excipients added. The aim of this study was to develop, characterize, and assess the drug release profiles of microcrystalline cellulose (MCC) and isomalt-based matrix pellets with direct drug incorporation, advancing beyond the earlier concept of isomalt as a mere inert core with layered drug application, under simulated physiological conditions in vitro. **Methods**: Matrix pellets with varying MCC–isomalt ratios (90:10, 70:30, and 50:50) were produced by extrusion/spheronization and subsequently coated with film-forming polymers. Dissolution experiments were performed under varying osmolarity to characterize release profiles. The experimental design data were statistically evaluated. **Results**: The extrusion/spheronization technique yielded uniform, robust matrix pellets with acceptable sphericity and mechanical integrity, even at high isomalt levels. Release from coated pellets was significantly influenced by the polymer coating and osmolarity of the dissolution medium. However, with increasing isomalt content in the matrix, the dependence of drug release kinetics on medium osmolarity was substantially reduced. **Conclusions**: The production of MCC–isomalt matrix pellets in which isomalt acts as a functional matrix component reduced the effect of osmolarity of dissolution medium on the release of ibuprofen sodium salt in in vitro experiments.

## 1. Introduction

Multiparticulate drug delivery systems based on pellets have attracted considerable interest in recent decades [1] as they offer distinct technological, physiological and therapeutic advantages over conventional single-unit dosage forms [2]. Pellets distribute the dose across numerous small units and thereby substantially reduce the risk of dose dumping since each individual unit releases only a limited amount of drug [3]. Within this context, the selection and design of suitable excipients for pellet cores and matrices are critical determinants of manufacturing performance and in vitro–in vivo behaviour.

Isomalt has emerged as a particularly versatile pharmaceutical excipient for oral multiparticulate systems, combining favourable technological functionality with patient-friendly properties. It is frequently employed as an inert core in multiparticulate dosage forms due to its excellent physicochemical characteristics and commercial availability in defined particle size fractions with varying ratios of glucopyranosyl mannitol (GPM) and glucopyranosyl sorbitol (GPS) [4]. Numerous studies have demonstrated that isomalt can enhance the release of selected active pharmaceutical ingredients and is especially suitable as an inert core in drug-layered pellet formulations [5,6,7,8,9]. Positron annihilation lifetime spectroscopy has revealed physical interactions between microcrystalline cellulose and isomalt in composite cores, indicating that increasing proportions of the water-soluble sugar alcohol intensify osmotic effects and thereby promote drug release [10].

In direct compression, isomalt is valued for its excellent flowability, high compressibility and low hygroscopicity, while its low glycaemic and insulinaemic responses, absence of relevant incompatibilities and function as a sugar substitute make it particularly suitable for formulations intended for diabetic patients [1,10]. Owing to its neutral taste, chemical stability, and pleasant mouthfeel, isomalt is particularly suitable as an excipient in chewable and rapidly disintegrating tablets.

Its agglomerated form exhibits high packing density and excellent compressibility, enabling its use in conventional tablets, as demonstrated by Grote et al. [11]. Furthermore, Lura et al. successfully applied agglomerated isomalt in combination with other excipients for the development of mini-tablets, orally dispersible tablets [12], while Bernard et al. used it in pellets [13]. It also functions effectively as a filler in 3D-printed dosage forms, where it provides advantageous sensory and mechanical properties [14]. In matrix pellets and other multiparticulates, isomalt enables optimised release characteristics [15], with its water solubility and chemical inertness contributing to constant release profiles in controlled release systems and allowing its use as a carrier for low- to high-dose formulations [5].

In contrast to conventional inert cores that are coated with solutions/suspensions/or melts of the active ingredient, matrix pellets represent an alternative formulation strategy in which the drug is incorporated directly into the matrix material. This approach allows uniform incorporation of substantially higher drug loads within the entire pellet structure [1,16] and offers two principal advantages: a homogeneous dispersion of the active substance and the possibility of achieving controlled release directly via the matrix itself [17,18]. More recently, the aspect of site-specific delivery, for example, for colon therapy [19], has been receiving increased attention through matrix-based multiparticulate systems. Studies have shown that matrix pellets can be developed on demand, without complex coatings, so that release is initiated only in the lower gastrointestinal tract, and systemic exposure is minimised [20]. In addition, matrix pellet systems offer a variety of other advantages, such as extending the half-life of active pharmaceutical ingredients [21] or compatibility with combination therapies [22] for improving patient convenience and compliance.

The release of active ingredients from matrix pellets is typically governed by several concurrent mechanisms, including diffusion, osmosis, and polymer erosion [23]. Other important factors affecting the release of active ingredients include the properties of the coatings (polymer types and coating thickness) and, notably, the properties of the core [24,25,26]. Among excipients, microcrystalline cellulose (MCC) is widely regarded as the gold standard for extrusion-spheronisation and is considered to have significant advantages over other materials. However, there are several disadvantages associated with its use, including the adsorption of active ingredients to the surface of its fibres [27], its chemical incompatibility with a number of active ingredients [28], and poor disintegration behaviour when used in matrix pellets [29].

In comparison with our previous study, where the release performance was evaluated from the mixture of layered isomalt and MCC composite cores [1], we aimed to examine the release pattern from matrix preparations, where the active pharmaceutical ingredient (API) was incorporated into an MCC–isomalt–API matrix monolithic pellet with a varied ratio of the excipients.

As a model compound, ibuprofen sodium, a non-steroidal anti-inflammatory drug (NSAID), is used. Due to its well-established pharmacological properties, e.g., high tolerability, extensively studied pharmacokinetics, short half-life, and high plasma protein binding, ibuprofen sodium salt serves as an ideal model drug for developing controlled-release systems, such as matrix pellets [17].

Matrix pellet systems are particularly sensitive to the osmotic effects due to their swellable polymer components and drug distribution throughout the matrix structure. The release mechanism involves dissolution medium penetration into the matrix, drug dissolution within the swollen pellet matrix, and subsequent diffusion through the hydrated polymer network. Each of these steps is influenced by the osmotic gradient between the pellet interior and the surrounding medium. The dependence of drug release on osmolarity was observed primarily in pellets with a high microcrystalline cellulose content, whereas pellets with a high isomalt content did not exhibit such dependence [30].

In addition to the properties of the core, the osmotic pressure in the gastrointestinal tract also plays an important role in the drug release profile of such pellets. The osmolality of gastrointestinal fluids varies considerably with nutritional state, ranging from approximately 29–276 mOsm/kg to a median of 559 mOsm/kg in the fasted stomach [31]. To reflect these postprandial conditions in a standardized in vitro setting, Jantratid et al. developed the Fed State Simulated Gastric Fluid (FeSSGF) with an osmolality of 400 mOsm/kg, representing the gastric environment between 75 and 165 min after meal intake [31,32,33].

These variations in the osmotic environment profoundly affect the mechanisms of drug release from matrix pellets through multiple pathways. Osmotic pressure gradients have been shown to drive water influx into pellet matrices, thereby creating internal pressure that has been demonstrated to influence drug dissolution and diffusion rates. Research has shown that an increase in osmolality in the release medium can have a substantial impact on the kinetics of drug release, with osmotic pressure proving to be a pivotal factor in the process of drug transport across intestinal barriers [34]. The composition of the release medium, which includes its pH, ionic strength and osmolarity, has been demonstrated to directly impact the swelling behaviour of polymer matrices [1,35].

The objective of this study was to investigate the applicability of matrix pellets based on MCC and isomalt and to compare the main characteristics of these formulations. Additionally, the study evaluated how varying matrix compositions and polymethacrylate copolymers with different permeability affect the in vitro release behaviour of ibuprofen sodium salt.

## 2. Materials and Methods

### 2.1. Materials

Ibuprofen sodium salt (Sigma-Aldrich Chemie GmbH, Merck, Darmstadt, Germany), galenIQ™ 800 (Beneo-Palatinit GmbH, Mannheim, Germany), further referred to as Isomalt and Vivapur^®^ 101 (JRS Pharma GmbH & Co., Rosenberg, Germany), further referred to as MCC, was used for pellet production. The matrix pellets were coated with Eudragit^®^ RL30D and Eudragit^®^ RS30D (Evonik Industries AG, Essen, Germany) polymers, Triethyl citrate (TEC; Fluka Chemie AG, Buchs, Switzerland) and micronized talc (Merck-Sigma-Aldrich Chemie GmbH, Darmstadt, Germany). The dissolution media were made of Disodium hydrogen phosphate (Molar Chemicals Ltd., Budapest, Hungary) and Sodium dihydrogenphosphate (Molar Chemicals Ltd., Budapest, Hungary). The osmolarity was increased with the use of glucose anhydrate (Molar Chemicals Ltd., Budapest, Hungary).

### 2.2. Preparation of MCC/Isomalt Matrix Pellets with Ibuprofen Sodium Salt

Ibuprofen sodium salt was used as a model drug. For matrix pellet preparation, isomalt and microcrystalline cellulose were mixed in three different weight ratios (MCC–isomalt 90:10, MCC–isomalt 70:30, and MCC–isomalt 50:50), to prepare a homogeneous excipient premix (Table 1). The mixture was then homogenized with 10%*w*/*w* ibuprofen sodium salt. The various powder blends were homogenized in a V-blender (CWV type; Xinxiang Chenwei Machinery Co., Ltd.; Xinxiang, China) at 105 rpm for 3 min (batch size: 150 g). The powder was then moistened with a sufficient amount of distilled water to form a plastic mass. The quantity of water is summarised in Table 1 (in the mass ratio relevant to the MCC + isomalt mixture). The moistened powder was extruded through a die (d = 1 mm) at 60 rpm using a single-screw rotating extruder (Caleva Multi Lab (CLM) 2014; Caleva Process Solutions Limited; Dorset, UK). The obtained extrudates were then rounded in a spheroniser (Locost GSZF-AK; Locost Kft.; Tiszaalpár, Hungary) over a cross-hatched plate for 3 min at 550 rpm. Finally, the pellets were dried in a drying chamber (WTC Binder Typ 17053099003100; BINDER GmbH, Tuttlingen, Germany) at 40 °C for 8 h. After drying, the pellets were sieved by a Retsch AS 200 vibration sieve (Retsch GmbH, Verder Scientific, Haan, Germany), and the fraction between 1.0 and 1.4 mm was used for all further experiments.

### 2.3. Determination of Loss on Drying

The residual water content of the matrix pellets was determined using the SCALTEC SMO 01 moisture analyser (SCALTEC Instruments GmbH, Heiligenstadt, Germany). The residual water content was determined by placing 10 g of pellets on the scale and testing them at 60 °C until mass consistency.

### 2.4. Coating Process

As film coating copolymers, Eudragit^®^ RL30D and Eudragit^®^ RS30D, further mentioned as ERL and ERS, were used. Triethyl citrate was used as a plasticizer, while micronized talc served as an anti-adhesion agent (Table 2). An amount of 3.08 mg/cm^2^ of coating polymer was applied to the pellets.

The variability in diameter and weight of the individual pellets was considered for each pellet composition. For the fluid bed coating, the Aeromatic STREA-1 (Aeromatic-Fielder AG, Bubendorf, Switzerland) was used. After the coating process (Table 3), the pellets were further dried for 10 min.

### 2.5. Size and Shape Analysis

The images taken by the Keyence VHX-970F digital microscope at 20× magnification (KEYENCE International NV/SA; Mechelen, Belgium) were utilised for image analysis. A total of 200 pellets were randomly selected from each batch for analysis. The pellets were positioned on a black background plate. One pixel is equal to 8.333 µm (120 pixels/mm). The resolution permitted capturing 20 pellets in a single frame. For each individual batch, a set of at least 10 photographs was taken and subsequently analysed using ImageJ (v.1.54g, National Institutes of Health, Bethesda, MD, USA). The dimensions and morphology of the pellets were characterised by a series of metrics, including roundness, aspect ratio (*AR*) and Feret diameter.

The area-equivalent spherical diameter (*d_eq_*) was related to the projection area of particles and was calculated based on the following equation (Equation (1)) [36]:

(1) $$d_{eq}=\sqrt{\frac{4\;A}{\pi }},$$

where A is the projection area.

The dimensions and morphology of the pellets were characterised by roundness, aspect ratio (*AR*), Feret diameter and area-equivalent diameter. The area-equivalent diameter (*d_eq_*) is used as the size measure, whereas the Feret diameters describe the largest extension of the particle and serve as the basis for the aspect ratio (Equation (2)):

(2) $$AR=\;\frac{major\;axis}{minor\;axis}$$

This parameter is a measure of the geometric shape of the pellet. A value closer to 1 indicates a more spherical and regular pellet shape, while deviations indicate oval or elongated shapes. For pellets, an *AR* value between 1.0 and 1.2 is considered optimal as this ensures an approximately spherical shape and the most homogeneous release of active ingredients possible [16,37]. Values outside this range indicate deformed or elongated pellets, which may adversely affect downstream processing steps such as capsule filling [38].

As a further shape descriptor, the roundness and the circularity were chosen. The roundness was calculated by the default software settings from the fitted ellipsis based on Equation (3):

(3) $$R=\frac{4\;A}{\pi \cdot {major\;axis\;}^{2}}$$

The circularity is more sensitive to surface irregularities. It was also calculated by the software based on Equation (4), where *C* stands for the circularity, *A* is the projection area and *P* is the Image J default corrected chain-code perimeter:

(4) $$C=\;\frac{4\cdot \pi \cdot A}{P^{2}}$$

### 2.6. Tensile Strength Test

To evaluate the tensile strength of the pellets, a sample of twenty pellets was randomly selected for testing. Each pellet is then positioned centrally on a steel plate within the CT3™ Texture Analyser (CT3-4500, Brookfield Engineering Laboratories, Middleboro, MA, USA), supplied with a 5 kg load cell. The upper cylinder (TA44, from the TA ProbeKit), with a diameter of 4 mm, is then lowered onto the pellet at a constant, controlled speed (0.1 mm/s). The force and diameter (*d*) of each individual pellet were registered. The application of maximum force (*F*) is automatically recorded as soon as the pellet begins to show signs of breakage or complete deformation. The surface tensile strength ($\sigma_{f})$ was calculated using the following equation (Equation (5)):

(5) $$\sigma_{f}\left(s\right)=\frac{1.6*F}{\pi *d^{2}}$$

All values are reported as mean ± SD (*n* = 20).

### 2.7. Structure Observation by Scanning Electron Microscope (SEM)

The scanning electron microscope images were processed by a Hitachi TM4000 Plus II Tabletop Scanning electron microscope (Hitachi, Tokyo, Japan). The samples were prepared and fixed on a double-sided conductive tape, and the pictures were taken in a standard vacuum mode at 5–15 kV accelerating voltage, lens mode 3 and a backscatter detector. The accelerating volume and magnification are indicated on the images.

### 2.8. Dissolution Study

The dissolution study was carried out using the rotating basket method (Hanson SR8-Plus™ Dissolution Test Station, Hanson Research, Chatsworth, CA, USA) at 50 rpm and 37.0 ± 0.5 °C in a 900 mL medium of pH 6.8 phosphate buffer. A sample corresponding to 0.08 g of coated pellets (*n* = 3), equivalent to 8 mg of ibuprofen sodium salt, was placed in each basket to ensure sink conditions, considering the API solubility. To evaluate drug release under varying osmotic conditions, glucose anhydrate (Molar Chemicals Kft., Budapest, Hungary) acted as the osmotically active component in the dissolution medium (glucose concentration: 0.00 mol/L glucose (Osm. I.), 0.275 mol/L (Osm. II.), and 0.550 mol/L (Osm. III.)). The osmolarity was determined by analysing 60 µL samples (*n* = 4) of the dissolution media using the Gonotec Osmomat 030 (Gonotec GmbH, Berlin, Germany). As a result of the added glucose, the osmolalities of the media used were as follows: Osm. I = 0.106 Osmol/kg, Osm. II = 0.483 Osmol/kg and Osm III = 0.706 Osmol/kg. At ten predetermined time points over a period of 8 h, the release of ibuprofen sodium was measured spectrophotometrically (Agilent 8453 UV-visible Spectroscopy System, Agilent Technologies, Inc. Headquarters, Santa Clara, CA, USA) at λ_ibuprofen_ = 222 nm. The three parallel measurements were performed within a time frame of at least three days and no more than seven days after coating each individual sample.

### 2.9. Pellet Release Kinetics and Statistical Analysis

Different dissolution profiles were observed during analysis of the pellets’ release kinetics. The Weibull distribution function was used to describe and compare these heterogeneous profiles [39,40]. This function is particularly well suited to characterising drug release processes where there is no uniform curve shape [41]. The Weibull equation is used to fit the release data (Equation (6)):

(6) $$M_{t}=M_{\infty }\left[1-exp{\left(-\frac{t-t_{0}}{\tau_{d}}\right)}^{\beta }\right]$$

*M_t_* represents the proportion of the active substance released until time *t*, starting at *t*_0_ initial time. $M_{\infty \;}$ represents the maximum release amount, *β* is the shape factor of the function and *t*_0_ represents the lag time. *τ_d_* stands for the characteristic time parameter (the time at which 63.2% of the active substance is released).

The effect of isomalt on pellet cores was evaluated according to a two-factor, three-level experimental design, based on dissolution parameters calculated according to the Weibull relationship. The coded values for the experimental setup are summarised in Table 4. The data were evaluated and graphically represented using TableCurve^®^ 3D v4.0 software (Systat Software Inc., London, UK).

A second-order polynomial model (Equation (7)) was used to determine the quantitative influence of the independent variables on the response variable y.

(7) $$y=b_{0}x_{0}+b_{1}x_{1}+b_{2}x_{2}+b_{11}x_{1}^{2}+b_{22}x_{2}^{2}+b_{12}x_{1}x_{2}$$

Here, *x*_1_ and *x*_2_ represent the factors under investigation, while the coefficients *b*_1_ and *b*_2_ represent the main linear effect, *b*_11_ and *b*_22_ represent the nonlinear quadratic and b_12_ represents the interaction effects. This model enabled the systematic investigation of the relationships between the formulation and environmental parameters and the release behaviour of the pellets.

## 3. Results

### 3.1. Loss on Drying

Prior to the mechanical tests, it was confirmed that the residual moisture content of all pellet formulations was between 2 and 3%, ensuring comparable moisture conditions for all samples.

### 3.2. Size and Shape Properties of Pellets

The values are the averages and standard deviations (±SD) calculated from 200 individual pellets (Table 5 and Table 6).

The average circularity values of the uncoated matrix pellets range between 0.84 and 0.86, which is within the range of good individual pellet morphology. The aspect ratio shows comparable values between 1.12 and 1.13 (Table 5). The shape parameters indicate that the pellets have a stable and uniform spherical character before and after coating (Table 5 and Table 6).

The equivalent diameters (*d_eq_*) show the size; the Feret diameters are utilized as a metric for the maximum expansion of the pellets. As illustrated in Table 5, the Feret values for the uncoated matrix pellets range from 1.12 to 1.34 mm, contingent upon the core type. Following the processes of coating, as illustrated in Table 6, higher Feret values from 1.17 to 1.41 mm were obtained, consistent with the expected outcomes.

### 3.3. Tensile Strength

In terms of mechanical strength of uncoated pellets (Table 5), the tensile strength increases as the proportion of isomalt in the matrix pellets increases. With the coating, an increase in tensile strength can be observed (Table 6). However, the variations within the groups remain relatively small.

### 3.4. SEM Images

**Figure 1:**
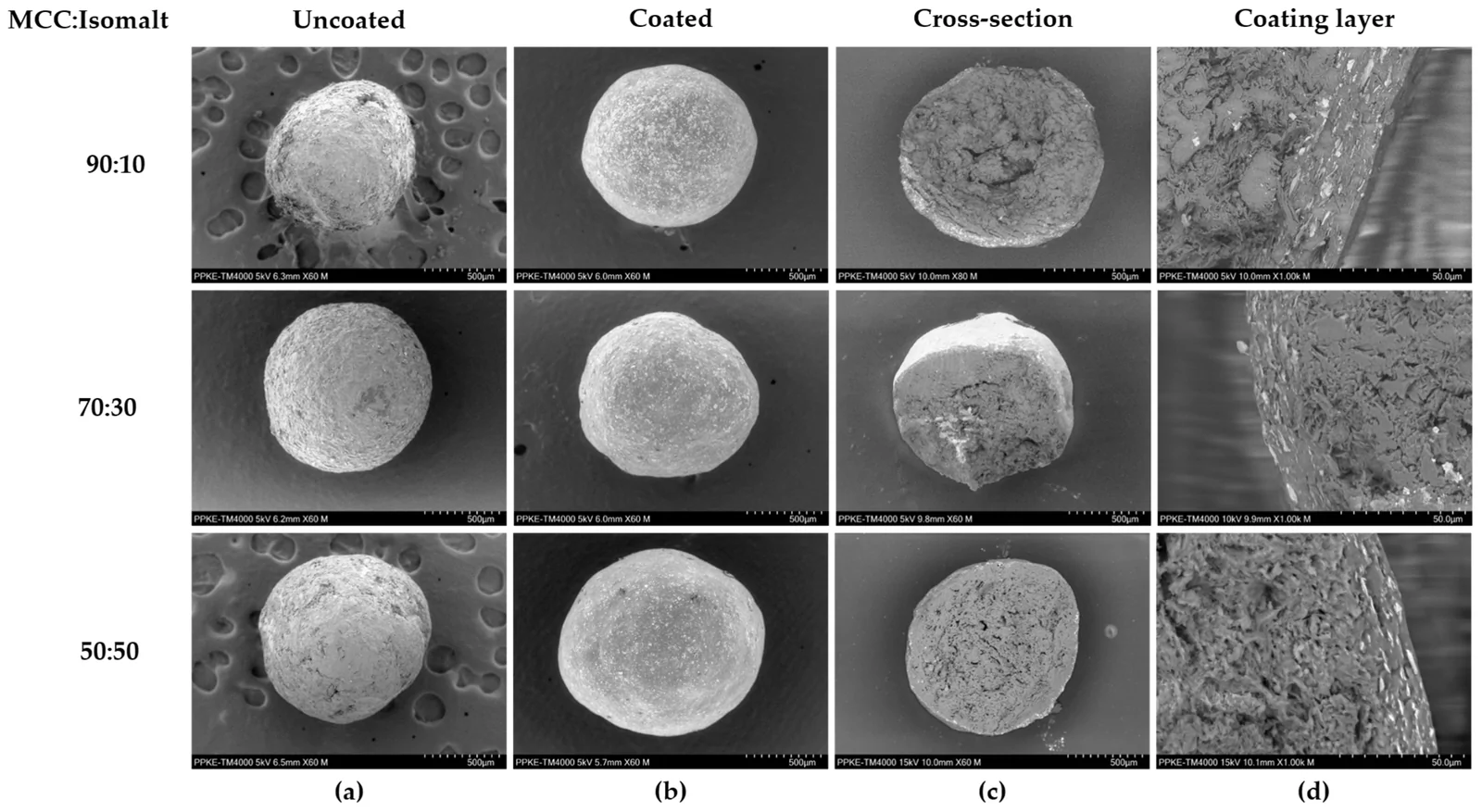
SEM images of ibuprofen sodium-containing matrix pellet cores of different composition (MCC–isomalt ratios 90:10, 70:30 and 50:50%*w*/*w*) before and after coating. Images show (**a**) the untreated matrix pellet core, (**b**) the ERL/ERS-coated pellets, (**c**) their cross section and (**d**) their coating layer. The images were taken with a Hitachi TM4000 Plus II (Hitachi Ltd., Tokyo, Japan). Scale bars indicate 500 μm (**a**–**c**) and 50 μm (**d**).

SEM analysis revealed distinct morphological characteristics of pellets manufactured with varying MCC–isomalt ratios (90:10, 70:30, and 50:50%*w*/*w*). The surface morphology and internal structure were evaluated for whole uncoated and coated pellets and cross-sectional views of coated samples (Figure 1).

SEM imaging of coated pellets revealed uniform, continuous coating layers with smooth external surfaces across all three formulations. The coating demonstrated consistent thickness and complete coverage without visible defects, cracks, or coating discontinuities at the examined magnifications. No significant morphological differences were observed between coating characteristics of the different core compositions, indicating that the MCC–isomalt ratio of the core material did not adversely affect coating application or adhesion.

Cross-sectional examination of cut pellets exposed the internal matrix structure and relationship to the pellet formulation. Compared with the compositions with higher MCC content, the formulations with higher isomalt content exhibited the most compact internal structure as the water-soluble isomalt content of the cores helped to fill the voids among the microcrystalline cellulose chains.

### 3.5. Dissolution Profile of Pellets

**Figure 2:**
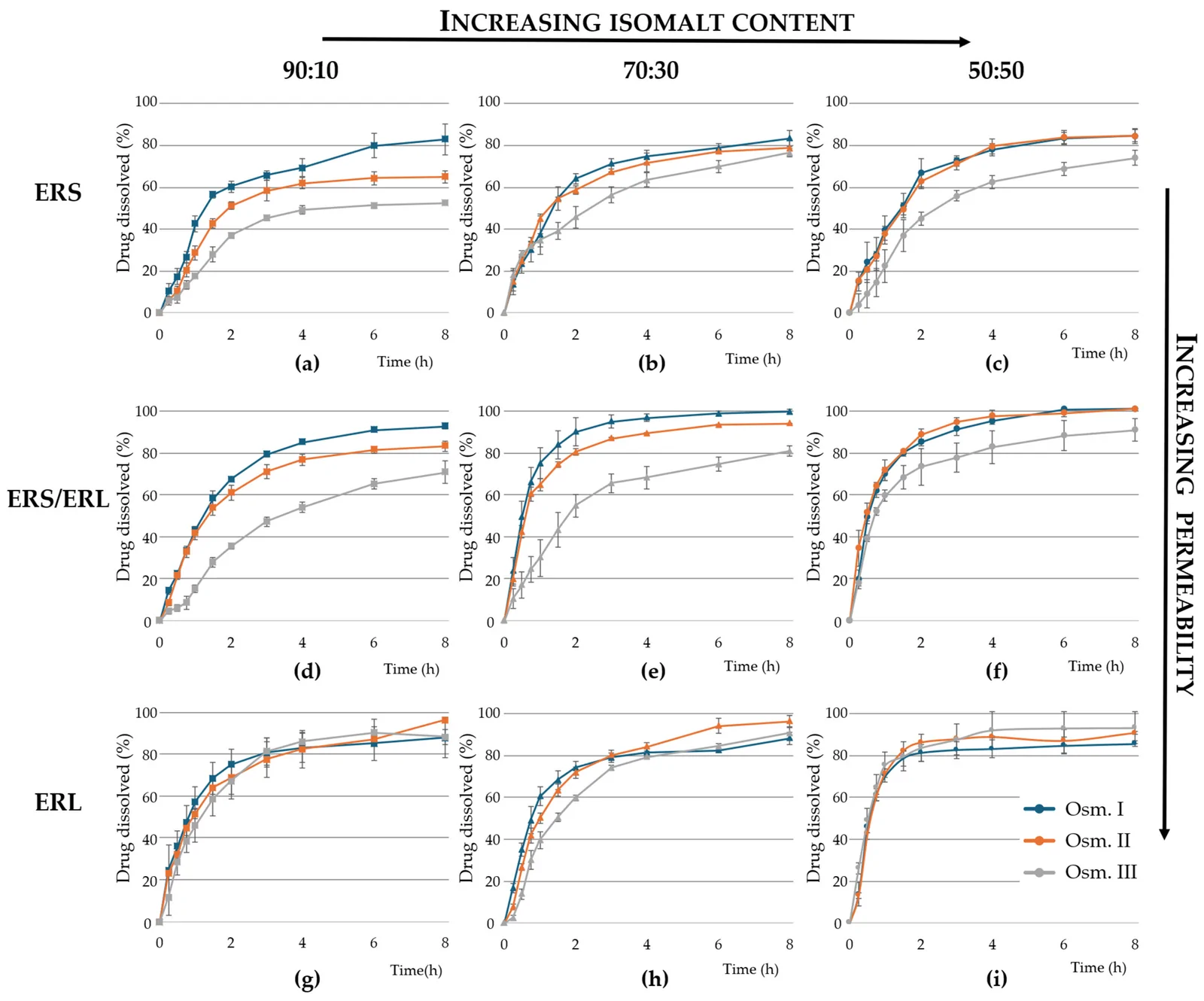
Drug release profiles of coated matrix pellets with different osmolarity, by addition of the following concentrations of glucose: 0.00 mol/L (Osm. I = 0.106 Osmol/kg) (blue), 0.275 mol/L (Osm. II = 0.483 Osmol/kg) (orange) and 0.550 mol/L (Osm. III = 0.706 Osmol/kg) (grey). Rows indicate the coating polymer and columns the MCC:Isomalt ratio (%w/w) of the pellet cores: (**a**) ERS, 90:10; (**b**) ERS, 70:30; (**c**) ERS, 50:50; (**d**) ERS/ERL, 90:10; (**e**) ERS/ERL, 70:30; (**f**) ERS/ERL, 50:50; (**g**) ERL, 90:10; (**h**) ERL, 70:30; (**i**) ERL, 50:50. Isomalt content in the matrix pellets increases from left to right, coating permeability increases from top to bottom. Data are shown as mean ± SD (*n* = 3).

The uncoated pellets performed immediate release of the API in all media. As illustrated in Figure 2, the release profile of ibuprofen from the three distinct matrix pellets varies with the three different coatings. An analysis of the release curves reveals that the dissolution is dependent not only on the pellet composition but also, to an equal extent, on the coating and the interaction between these two factors.

The permeability of the coatings influenced the release performance. Eudragit^®^ RL coating is the most permeable, leading to faster release (Figure 2g–i). Eudragit^®^ RS has lower permeability, thereby slowing the release (Figure 2a–c). The release curves of the coating mixture of Eudragit^®^ RS and RL in a 1:1 ratio lie between the two extremes (Figure 2d–f). Where the coating permeability was higher (ERL), the effect of the osmotic environment was less pronounced than for the coatings of lower permeability (ERS and ERS/ERL; Figure 2a–f). Furthermore, the effect of osmolarity was greater in the pellets with higher MCC content (90:10 and 70:30).

The pellet composition influences the release profiles depending on the coating polymer composition (Figure 2). The difference in dissolution curves between Osm. I, II and III is evident with the higher MCC content in the pellets and declining coating permeability.

### 3.6. Release Kinetics of Pellets

The two-factor, three-level design and the $\tau_{d}$ (h) values obtained from the Weibull distribution function are compiled in Table 7. $\tau_{d}$ (h) denotes the time at which 63.2% of ibuprofen sodium salt has been released from the matrix pellets. The Weibull fits reproduced the experimental release curves with high correlation (r ≥ 0.972 throughout).

**Figure 3:**
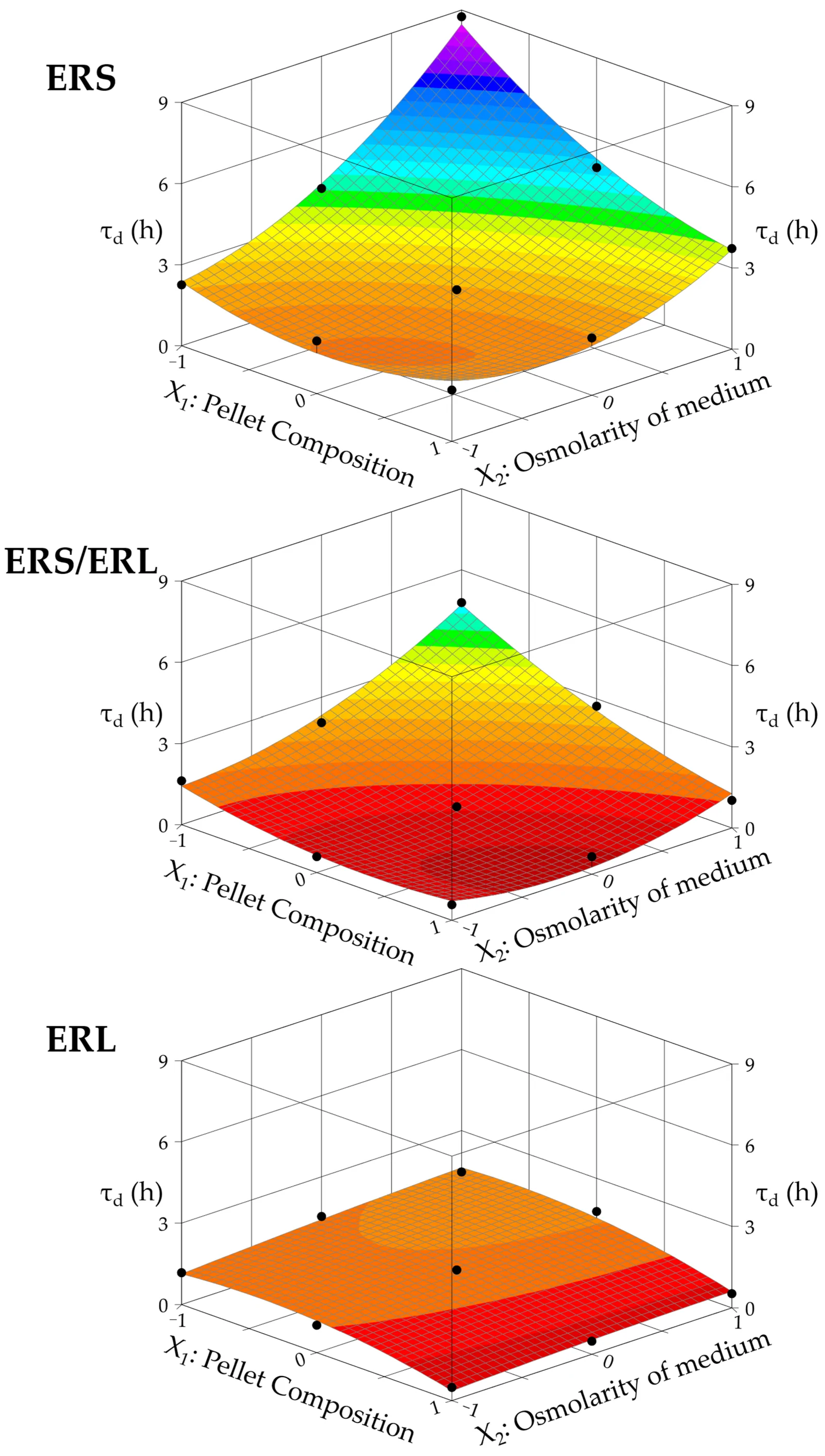
Characteristic time value deduced from the Weibull distribution analysis. A surface plot shows the effects of pellet composition and osmolarity on dissolution, sorted by the different polymer coatings. (*τ_d_* demonstrates characteristic time value when 63.2% of drug is dissolved.) The colour gradient represents the magnitude of the fitted values, with red/orange indicating lower values and blue/purple indicating higher values.

The surface plots (Figure 3) illustrate the dependence of $\tau_{d}$ on pellet composition and osmolarity for the three coatings.

The influence of the examined factors on $\tau_{d}$ was statistically significant for the ERS and ERS/ERL coatings and non-significant for the ERL coating (Table 8). For the ERS coating the regression was highly significant (F = 36.884; *p* > 0.0068). A positive regression coefficient indicates that an increase in the corresponding independent variable prolongs the time $\tau_{d}$ required to release 63.2% of the active ingredient.

(8) $$\tau_{d,ERS}=2.253-1.230x_{1}+1.885x_{2}+1.130x_{2}^{2}-1.169x_{1}x_{2}\;\left(\mathrm{with}\;r_{ERS}=0.984,\mathrm{fitted}\mathrm{Std}\mathrm{Err}=0.46\right)$$

(9) $$\tau_{d,ERS/ERL}=-1.038x_{1}+0.959x_{2}-0.682x_{1}x_{2}\;\left(\mathrm{with}\;r_{ERS/ERL}=0.975,\mathrm{fitted}\mathrm{Std}\mathrm{Err}=0.36\right)$$

A polynomial equation (Equation (7)) is applied to describe the effects of the independent variables. The pellet composition (*x*_1_) of the ERS coating demonstrates a substantial negative linear effect (*b*_1_ = −1.230; *p* > 0.007), so that an increasing isomalt fraction shortens the release time (lower *τ_D_*). Conversely, the osmolarity of the medium (*x*_2_) has a significant positive influence (*b*_2_ = 1.885; *p* > 0.002), with a higher osmolarity leading to a longer release time. For the osmolarity a significant quadratic effect was found (*b*_22_ = 1.130; *p* > 0.041), indicating that the dependence on osmolarity is not purely linear but passes through an optimum in the range investigated. The quadratic term of the pellet composition (*b*_11_ = 0.803; *p* > 0.092) was not statistically significant and thus was eliminated from the reduced model equation (Equation (8)). The interaction effect was significantly negative (*b*_12_ = −1.169; *p* > 0.015; Equation (8)). The positive sign of *b*_2_ reflects an osmotic-induced prolongation of the release time. Glucose in the dissolution medium reduces the osmotic gradient between the pellet interior and the bulk medium, so that water uptake through the film decreases. Slower hydration of the core delays dissolution of the drug within the pellet and lowers the concentration gradient across the membrane, which prolongs $\tau_{d}$.

The polynomial model describing the release by the ERS/ERL coating is also significant (F-value 23.037; *p* > 0.0134), with an increase in isomalt content showing a faster release time (*b*_1_ = −1.038; *p* > 0.006) and a higher osmolarity leading to a slower release process (*b*_2_ = 0.959; *p* > 0.008). Despite the positive square terms for both factors, these are not significant (*b*_11_: *p* > 0.187 ^†^; *b*_22_: *p* > 0.064 ^†^), in contrast to the ERS coating. The interaction effect remained significantly negative, although less pronounced than for the ERS coating (*b*_12_ = −0.682; *p* > 0.033). The interaction coefficient (*b*_12_) was significant for the ERS and ERS/ERL coatings but not for the ERL coating, indicating a coupling between pellet composition and osmolarity in the coatings of lower permeability (Equation (9)).

The model fit for the ERL coating was non-significant (F-value 7.113; *p* > 0.0686 ^†^). Owing to the high permeability of this coating and the resulting rapid release, neither pellet composition nor osmolarity exerted a significant effect on $\tau_{d}$.

**Figure 4:**
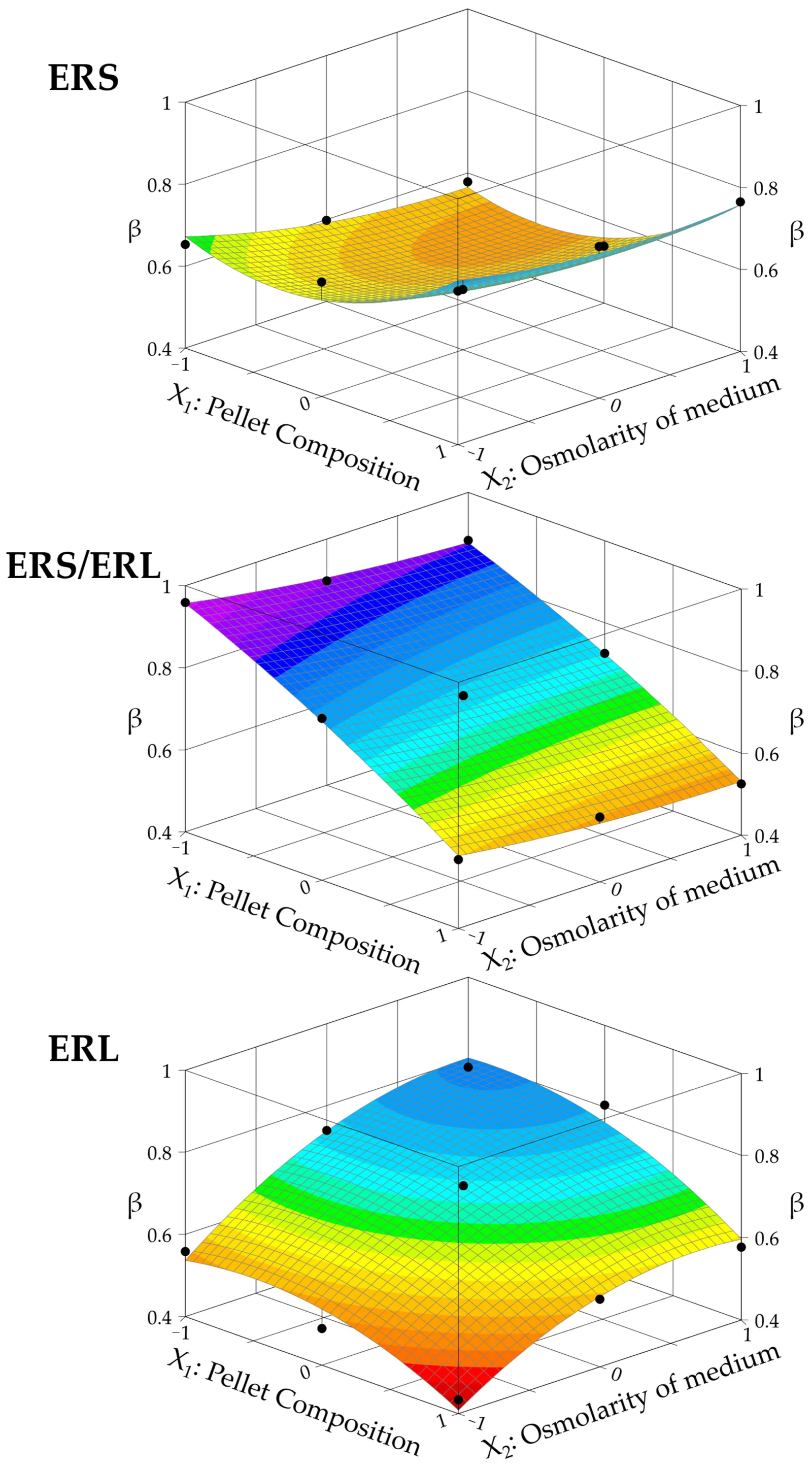
Shape parameter (*β*) of release curves based on dissolution Weibull analysis, illustrating the effects of pellet composition and osmolarity on the drug release mechanism, sorted according to the different polymer coatings. (*β*) describes the shape of the release profile. The colour gradient represents the magnitude of the fitted values, with red/orange indicating lower values and blue/purple indicating higher values.

The effects of the factors considered on the Weibull shape parameter *β* are different for the three coatings, but all remain below 1 (Table 9). The surface plots fitted according to the polynomial model (Equation (7)) are summarized in Figure 4.

(10) $$\beta_{,ERS}=0.571+0.080x_{1}+0.101x_{1}^{2}\;\left(\mathrm{with}\;r_{ERS}=0.958,\mathrm{fitted}\mathrm{Std}\mathrm{Err}=0.04\right)$$

(11) $$\beta_{,ERS/ERL}=0.744-0.181x_{1}-0.031x_{2}\;\left(\mathrm{with}\;r_{ERS/ERL}=0.997,\mathrm{fitted}\mathrm{Std}\mathrm{Err}=0.01\right)$$

(12) $$\beta_{,ERL}=0.717-0.083x_{1}+0.113x_{2}\;\left(\mathrm{with}\;r_{ERL}=0.939,\mathrm{fitted}\mathrm{Std}\mathrm{Err}=0.04\right)$$

A significant regression model was obtained for the ERS coating (F = 9.278; *p* > 0.0481). Pellet composition showed both a significant positive linear effect (*b*_1_ = +0.080; *p* > 0.015) and a significant positive quadratic effect (*b*_11_ = +0.101; *p* > 0.034). (Equation (10)) A higher isomalt content thus increased the beta value and shifted the release profile toward a combined mechanism of Fickian diffusion and Case II transport. The effect of osmolarity is negative but not significant (*b*_2_ = −0.037; *p* > 0.099), meaning that beta values tend to decrease as osmolarity increases. For all pellet formulations coated by the ERS coating layer, the beta value decreases from osmolarity I to osmolarity III.

For the ERS/ERL coating, the model is the most pronounced among all three systems (F = 216.852; *p* > 0.0005). Both pellet composition and osmolarity show a clear negative linear effect (*b*_1_ = −0.181; *p* > 0.001) (*b*_2_ = −0.031; *p* > 0.011). (Equation (11)) Quadratic and interaction effects are not significant. Thus, both a higher isomalt content and a higher osmolarity of the release medium lead to lower β values and shift the release profile toward a Fickian diffusion behaviour.

A significant regression model can also be demonstrated for the ERL coating (F = 13.725; *p* > 0.0280). Pellet composition shows a significant negative linear effect (*b*_1_ = −0.083; *p* > 0.020), while osmolarity exhibits a significant positive effect (*b*_2_ = +0.113; *p* > 0.008). (Equation (12)) Quadratic and interaction terms are not significant.

## 4. Discussion

### 4.1. Size and Shape Evaluation of Pellets

The physical and chemical properties of pellets, such as their shape, surface geometry, and size, are crucial for the quality and release of the dosage form.

Kleinebudde et al. evaluated matrix pellets based on their aspect ratio; shape was used as the quality parameter [37]. Kranz et al. described that the aspect ratio (*AR*) is considered acceptable between 1 and 1.2, with 1 describing an ideal sphere [16,37]. Our results from *AR* ranges from 1.10 to 1.18 and *R* ranges from 0.85 to 0.91 fall within the range specified by Kranz et al. [16].

There is a variation in size distribution, with a Feret diameter ranging from 1.12 to 1.34 mm for uncoated and 1.17 to 1.41 mm for coated pellets (Table 5 and Table 6). Akpabio et al. [42] investigated the influence of granule size on release kinetics of ibuprofen in matrix tablets and found that a mixture of particles of different sizes did not influence the release kinetics with statistical significance. The coating process can be effectively performed on the surfaces of all the pellet compositions, indicated by the shape parameters *AR*, *C* and *R* also remaining largely unchanged during the coating process. This is not illustrated solely by the shape parameters but also by the low standard deviation, further confirming the very low variability in the shape of the pellets.

The increase in tensile strength with increasing isomalt content, together with the compact internal structure seen in the cross-sectional SEM images, is consistent with a denser matrix.

### 4.2. Influence of the Pellet Composition on the Release

The results prove that the composition of the pellet cores significantly influences the release profile. Pellets with a high proportion of isomalt in their composition release the model active ingredient faster than pellets with a higher MCC content. Kállai et al. [1] have also described that the drug layered on highly water-soluble sugar or isomalt-based inert pellets release the model active ingredient quickly. This steeper, faster release can be explained by isomalt’s higher solubility (25 g/100 mL solution at 20 °C) [2,30]. In contrast, pellets with a higher MCC content exhibit delayed and incomplete release behaviour.

### 4.3. The Effect of Dissolution Medium Osmolarity on the Release of Active Ingredient

The osmolarity of the medium has a considerable influence on the release of the active ingredient, although the influence of the coating polymer is dominant. The release curves (Figure 2) become flatter with increasing osmolarity. It has been demonstrated that as osmolarity increases, both the initial release rate is slowed down and the maximum release in 8 h is lower. The effect is particularly pronounced in matrix systems with osmotically active cores, as Muschert et al. (2010) also demonstrated on ethylcellulose-coated pellets [43]. Mechanistically, the reduced osmotic gradient between the pellet core and the surrounding medium decreases the rate of water influx and thereby slows release [1].

This behaviour is consistent with the classic finding of Ozturk et al. [44], who showed for ethylcellulose-coated pellets that the release rate is inversely related to the osmotic pressure of the medium, indicating an osmotically driven contribution to release superimposed on diffusion. A clear correlation was also observed between the magnitude of the osmolarity effect and the permeability of the film coating. For low-permeability ERS coatings, the effect of different osmolarities is more pronounced than for high-permeability ERL coatings.

### 4.4. Kinetic Analysis of In Vitro Drug Release from Matrix Pellets

The release data were analysed using the Weibull model (Equation (6)), and the effects of the independent variables were quantified using a second-order polynomial model (Equation (7)). Two target variables were considered: the characteristic time $\tau_{d}$ as a measure of the release rate and the shape parameter β, which, according to Papadopoulou et al. [41], allows conclusions to be drawn about the transport mechanism (β≤0.75, Fickian diffusion; 0.75<β<1, a combined mechanism involving Fickian diffusion and Case II transport; and β >1, a complex mechanism).

For the statistical analysis of the $\tau_{d}$, the following conclusions can be drawn from the data. For coatings containing ERS, an increasing MCC content prolongs the release time, as does a higher osmolarity of the medium. The significantly negative coefficient $b_{12}$ shows an antagonistic interaction between the two factors. The inhibitory effect of osmolarity decreases as the isomalt content increases. The significant quadratic term for osmolarity ($b_{22}=+1.130$) also points out that within the range studied, the osmolarity-dependence exhibits saturation behavior. In the ERS/ERL coating, the same linear trends and the same antagonistic coupling were observed but without significant curvature, whereas in the permeable ERL coating, neither of these factors had a discernible effect on $\tau_{d}$. Muschert et al. [45] reached a similar conclusion, namely, that the release kinetics of coated pellets result from the interaction between core composition and medium osmolarity and not from either factor alone.

All three β-models from the statistical analysis are significant. With all values below 1, the profiles fall between pure Fickian diffusion and a combined Fickian/Case II mechanism. Eudragit^®^ RS and RL are water-insoluble, pH-independent, and non-erodible in their swollen state [46].

It is revealing that the osmolarity effect has the opposite sign on β for both types of membranes. For the low-permeability ERS and ERS/ERL coatings, the β value decreases. In contrast, for ERL coatings with high permeability, the β value increases as osmolarity rises. This reversal can be explained by the different role of osmotically driven water uptake, the extent of which decreases in any case as soon as the external osmolarity increases and the osmotic gradient decreases [44]. In the case of less water-permeable membranes, the film constitutes the rate-limiting resistance. A large osmotic gradient drives water through the film with a lower permeability and builds up an internal pressure that supplements the slow membrane diffusion with a delayed, osmotic effect, β shifts toward the combined region. Higher osmolarity in the dissolution media suppresses this driving force, and the release is reduced to membrane-limited Fickian diffusion (β decreases below 1). With the ERL membrane, the opposite is true since the film offers a lower resistance, and the release is determined by the hydration and dissolution of the core. The strong influx of water at low osmolarity produces a pronounced initial release. If the osmolarity is increased, this initial release is dampened and distributed more evenly over time, resulting in β rising.

The compositional effect on β fits into this picture. With the less permeable ERS coating, an increase in isomalt content raises the β value. The core becomes more osmotically active and absorbs more water. However, the low-permeability membrane limits the influx of release medium, so that the core’s hydration proceeds only gradually. As a result, the release is delayed and has a less pronounced initial release, which results in a higher β value. For the ERL and ERS/ERL coatings, an increasing isomalt content reduces β since the more soluble core accelerates the early release, which dominates when the membrane is not the rate-limiting step.

$\tau_{d}$ describes the extent of prolongation and is determined by the antagonistic coupling between the osmotically active isomalt core and the external medium osmolarity. β, by contrast, indicates whether release is characterised by gradient-driven diffusion or by a slower process due to the osmotic effect of the core.

Since the coating membranes are not eroding under the applied conditions, this release mechanism can be attributed to the osmotic activity of the core, and its expression is governed by the film permeability. With the permeable ERL coating, the core effect becomes fully apparent, with the higher osmolarity medium attenuating the initial release and raising β. With the less permeable coatings, it is gated by the membrane, with the higher osmolarity release environment suppressing the osmotic effect of the core, accompanied by a non-significant trend toward lower β.

In summary, it can be said that an increasing concentration of isomalt in the pellet matrix reduces the effect of increased osmolarity in the medium. The kinetic Weibull analysis confirms that targeted variation in the matrix composition and osmolarity of the medium is crucial to achieve controlled-release profiles in pellet-based drug forms.

## 5. Conclusions

This study demonstrates the development of novel matrix pellets with a significant proportion of isomalt as a functional additive. Earlier methods were primarily reliant on inert composite pellet cores, where isomalt functioned exclusively as a starter core. The developed matrix pellets demonstrate adequate sphericity and mechanical integrity across all composition ratios investigated (90:10, 70:30, and 50:50), with the extrusion/spheronization technology even at high isomalt values.

Matrix pellets show reduced dependence on medium osmolarity with increasing isomalt content considering the dissolution profile, which was particularly evident in the case of low-permeability coatings. This property differentiates the novel high-isomalt matrix pellets from conventional inert-coated cores, presenting significant advantages for developing robust, predictable release profiles under varying physiological conditions and opening new possibilities for patient- and indication-specific therapeutic approaches with consistent drug release.

## Figures and Tables

**Table 1:** Mass ratio of excipients used for the preparation of ibuprofen sodium-loaded matrix pellet.

Code of Formula	Solid Components of the Excipient Premix		Water for Pelletisation
	MCC	Isomalt	
90:10	9	1	9
70:30	7	3	6
50:50	5	5	4

**Table 2:** Composition of film coating dispersions used for the coating of MCC–isomalt–API matrix pellets (%*w*/*w*).

Component	ERS	ERS–ERL	ERL
Eudragit^®^ RS 30D	33.30%	16.65%	-
Eudragit^®^ RL 30D	-	16.65%	33.30%
Triethyl citrate (TEC)	2.00%	2.00%	2.00%
Talcum	7.50%	7.50%	7.50%
Water	57.20%	57.20%	57.20%

**Table 3:** Parameters of Aeromatic STREA-1, for coating with Eudragit^®^ polymers.

Setting Parameters of Aeromatic STREA-1	Values
Batch size (g)	150.0
Spray nozzle diameter (mm)	0.8
Inlet air temperature (°C)	44.0–47.0
Set temperature (°C)	45.0
Outlet air temperature (°C)	39.0–41.0
Atomizing air pressure (bar)	1.0
Fluid air stage	1.0–4.5
Fluid air flow rate (m^3^/h)	70–80
Spray rate (g/min)	2.0–3.5
Drying temperature (°C)	45.0
Drying time (min)	10.0

**Table 4:** Experimental setup, independent variables of pellet composition, glucose content of the medium and their code values.

	Actual Values	
Coded Value	*X*_1_, Pellet Excipient Composition (MCC–Isomalt Mass Ratio)	*X*_2_, Glucose Concentration of Dissolution Medium (mol/L)
−1	90:10	0.000
0	70:30	0.275
+1	50:50	0.550

**Table 5:** Physical and morphological properties of uncoated matrix pellets with varying core compositions (mean ± SD).

Excipient Ratio of Matrix Pellet Core (%*w*/*w*) (MCC–Isomalt)	Tensile Strength (N/mm^2^)	Feret Diameter (mm)	*d_eq_* (mm)	Shape Parameters		
				AR	Roundness	Circularity
90:10	2.24 ± 0.91	1.12 ± 0.09	1.03 ± 0.08	1.12 ± 0.08	0.89 ± 0.06	0.84 ± 0.04
70:30	2.75 ± 0.84	1.29 ± 0.10	1.18 ± 0.09	1.13 ± 0.07	0.88 ± 0.06	0.85 ± 0.03
50:50	4.01 ± 0.84	1.34 ± 0.14	1.23 ± 0.10	1.13 ± 0.10	0.88 ± 0.07	0.86 ± 0.03

**Table 6:** Physical and morphological properties of coated matrix pellets with varying core compositions (mean ± SD).

	Coating Layer		Tensile Strength (N/mm^2^)	Feret Diameter (mm)	*d_eq_* (mm)	Shape Parameters		
Core Type (MCC– Isomalt)	ERS (%)	ERL (%)				AR	Roundness	Circularity
90:10	100	0	2.41 ± 0.82	1.18 ± 0.13	1.08 ± 0.09	1.12 ± 0.08	0.89 ± 0.06	0.88 ± 0.08
	50	50	3.60 ± 0.81	1.18 ± 0.12	1.09 ± 0.10	1.12 ± 0.08	0.89 ± 0.01	0.87 ± 0.02
	0	100	3.39 ± 1.03	1.17 ± 0.13	1.09 ± 0.12	1.18 ± 0.10	0.85 ± 0.07	0.88 ± 0.02
70:30	100	0	3.36 ± 1.42	1.36 ± 0.16	1.25 ± 0.14	1.13 ± 0.07	0.89 ± 0.05	0.85 ± 0.03
	50	50	4.13 ± 1.83	1.36 ± 0.13	1.25 ± 0.12	1.13 ± 0.07	0.89 ± 0.07	0.87 ± 0.02
	0	100	3.28 ± 0.79	1.37 ± 0.14	1.26 ± 0.12	1.12 ± 0.06	0.89 ± 0.05	0.86 ± 0.02
50:50	100	0	4.17 ± 1.14	1.41 ± 0.20	1.29 ± 0.17	1.14 ± 0.08	0.88 ± 0.06	0.85 ± 0.03
	50	50	4.17 ± 0.58	1.41 ± 0.16	1.32 ± 0.14	1.10 ± 0.07	0.91 ± 0.05	0.87 ± 0.02
	0	100	4.12 ± 0.85	1.40 ± 0.17	1.31 ± 0.15	1.11 ± 0.06	0.90 ± 0.05	0.87 ± 0.02

**Table 7:** Two-factor matrix comprising three coatings using the Weibull distribution function-based estimation of kinetic parameters of dissolution.

Coating Polymer	Trial Number	Experimental Matrix		$\tau_{d}$ (z) (h)	*β*	*t*_0_ (h)	*R*
		*X*_1_ (x)	*X*_2_ (y)				
ERS	1	−1	−1	2.256 ± 0.617	0.653 ± 0.155	0.204 ± 0.033	0.986
	2	−1	0	4.112 ± 0.071	0.598 ± 0.023	0.227 ± 0.010	0.972
	3	−1	1	8.752 ± 0.564	0.578 ± 0.038	0.219 ± 0.012	0.974
	4	0	−1	1.945 ± 0.061	0.679 ± 0.157	0.164 ± 0.014	0.989
	5	0	0	2.135 ± 0.221	0.547 ± 0.024	0.156 ± 0.075	0.990
	6	0	1	4.941 ± 0.578	0.539 ± 0.051	0.044 ± 0.062	0.996
	7	1	−1	1.899 ± 0.303	0.775 ± 0.117	0.112 ± 0.046	0.991
	8	1	0	2.119 ± 0.269	0.770 ± 0.002	0.096 ± 0.023	0.993
	9	1	1	3.720 ± 0.520	0.765 ± 0.079	0.214 ± 0.050	0.991
ERS/ERL	1	−1	−1	1.620 ± 0.147	0.959 ± 0.045	0.041 ± 0.058	0.999
	2	−1	0	2.065 ± 0.267	0.898 ± 0.013	0.028 ± 0.020	0.993
	3	−1	1	4.790 ± 0.351	0.883 ± 0.013	0.230 ± 0.028	0.990
	4	0	−1	0.587 ± 0.164	0.794 ± 0.279	0.140 ± 0.080	1.000
	5	0	0	0.727 ± 0.037	0.736 ± 0.002	0.139 ± 0.025	0.997
	6	0	1	2.728 ± 0.573	0.725 ± 0.098	0.168 ± 0.012	0.993
	7	1	−1	0.578 ± 0.072	0.568 ± 0.048	0.210 ± 0.004	0.998
	8	1	0	0.647 ± 0.021	0.558 ± 0.003	0.120 ± 0.068	0.997
	9	1	1	1.019 ± 0.154	0.525 ± 0.167	0.205 ± 0.028	0.998
ERL	1	−1	−1	1.171 ± 0.282	0.558 ± 0.072	0.137 ± 0.098	0.993
	2	−1	0	1.535 ± 0.486	0.739 ± 0.070	0.026 ± 0.022	0.994
	3	−1	1	1.468 ± 0.363	0.780 ± 0.103	0.140 ± 0.102	0.997
	4	0	−1	1.007 ± 0.228	0.489 ± 0.040	0.214 ± 0.021	0.993
	5	0	0	1.333 ± 0.164	0.722 ± 0.014	0.210 ± 0.013	0.998
	6	0	1	1.778 ± 0.149	0.805 ± 0.059	0.237 ± 0.009	0.997
	7	1	−1	0.485 ± 0.083	0.434 ± 0.104	0.240 ± 0.011	0.994
	8	1	0	0.482 ± 0.018	0.564 ± 0.012	0.235 ± 0.003	0.993
	9	1	1	0.526 ± 0.137	0.577 ± 0.167	0.186 ± 0.033	0.996

**Table 8:** Summary of statistical analysis of the dissolution parameter ($\tau_{d}$) by the Weibull model.

Coating Polymer	Model F-Value	Parameter	Coefficients					
			*b*_0_	*b*_1_	*b*_2_	*b*_11_	*b*_22_	*b*_12_
ERS	36.884 (*p* > 0.0068)	Value	2.253	−1.230	1.885	0.803	1.130	−1.169
		Standard error	0.346	0.189	0.189	0.328	0.328	0.232
		*p* > |*t*|	0.007	0.007	0.002	0.092 ^†^	0.041	0.015
ERS/ ERL	23.037 (*p* > 0.0134)	Value	0.853	−1.038	0.959	0.439	0.741	−0.682
		Standard error	0.272	0.149	0.149	0.258	0.258	0.182
		*p* > |*t*|	0.052 ^†^	0.006	0.008	0.187 ^†^	0.064 ^†^	0.033
ERL	7.113 (*p* > 0.0686 ^†^)	Value	1.402	−0.447	0.185	−0.428	−0.044	−0.064
		Standard error	0.167	0.091	0.091	0.158	0.158	0.112
		*p* > |*t*|	0.003	0.016	0.136 ^†^	0.074 ^†^	0.798 ^†^	0.608 ^†^

^†^ Not significant.

**Table 9:** Summary of statistical analysis of the Weibull shape parameter (*β*) describing the drug release mechanism as a function of pellet composition and osmolarity.

Coating Polymer	Model F-Value	Parameter	Coefficients					
			b_0_	b_1_	b_2_	b_11_	b_22_	b_12_
ERS	9.278 (*p* > 0.0481)	Value	0.571	0.080	−0.037	0.101	0.026	0.016
		Standard error	0.029	0.016	0.016	0.027	0.027	0.019
		*p* > |*t*|	0.000	0.015	0.099 ^†^	0.034	0.406 ^†^	0.464 ^†^
ERS/ ERL	216.852 (*p* > 0.0005)	Value	0.744	−0.181	−0.031	−0.020	0.012	0.008
		Standard error	0.010	0.006	0.006	0.010	0.010	0.007
		*p* > |*t*|	0.000	0.001	0.011	0.134 ^†^	0.316 ^†^	0.316 ^†^
ERL	13.725 (*p* > 0.0280)	Value	0.717	−0.083	0.113	−0.063	−0.068	−0.020
		Standard error	0.033	0.183	0.183	0.032	0.032	0.022
		*p* > |*t*|	0.000	0.020	0.008	0.140 ^†^	0.122 ^†^	0.443 ^†^

^†^ Not significant.

## Data Availability

Measurement data are available upon request.

## Abbreviations

The following abbreviations are used in the manuscript:

A	Area
API	Active pharmaceutical ingredient
AR	Aspect ratio
b_0_	Constant term of the polynomial model
b_1_, b_2_	Linear regression coefficients of x_1_ (pellet composition) and x_2_ (osmolarity)
b_11_, b_22_	Quadratic regression coefficients of x_1_ (pellet composition) and x_2_ (osmolarity)
b_12_	Interaction coefficient
C	Circularity
d	Diameter
d_eq_	Area-equivalent spherical diameter
ERL	Eudragit^®^ RL30D
ERS	Eudragit^®^ RS30D
F	Force (maximum)
FeSSGF	Fed-state simulated gastric fluid
GPM	Glucopyranosyl mannitol
GPS	Glucopyranosyl sorbitol
MCC	Microcrystalline cellulose
mOsm	Milliosmole
M_t_	The percentage of drug dissolved at time *t*
$M_{\infty }$	Maximum release
n	Number of replicates
NSAID	Non-steroidal anti-inflammatory drug
*p*	Probability value
P	Perimeter
r	Correlation coefficient
R	Roundness
SD	Standard deviation
SEM	Scanning electron microscope
t	Time
t_0_	lag time
TEC	Triethyl citrate
τ_d_ (h)	Time when 63.2% of the drug is dissolved
UV	Ultraviolet
x_1_	Coded value of the pellet excipient composition
x_2_	Coded value of the glucose concentration of the dissolution medium
y	Response variable of the polynomial model
β	Shape factor of the Weibull function
σ_f_	Surface tensile strength
